# Modelling the expected probability of correct assignment under uncertainty

**DOI:** 10.1038/s41598-020-71558-x

**Published:** 2020-09-15

**Authors:** Tom Dvir, Renana Peres, Zeév Rudnick

**Affiliations:** 1 Racah Institute of Physics, The Hebrew University, 91904 Jerusalem, Israel; 2 School of Business Administration, Hebrew University of Jerusalem, 91905 Jerusalem, Israel; 3 School of Mathematical Sciences, Tel Aviv University, 69978 Tel Aviv, Israel; 4 QuTech and Kavli Institute of Nanoscience, Delft University of Technology, 2600 GA Delft, The Netherlands

**Keywords:** Applied mathematics, Information theory and computation, Statistical physics

## Abstract

When making important decisions such as choosing health insurance or a school, people are often uncertain what levels of attributes will suit their true preference. After choice, they might realize that their uncertainty resulted in a mismatch: choosing a sub-optimal alternative, while another available alternative better matches their needs. We study here the overall impact, from a central planner’s perspective, of decisions under such uncertainty. We use the representation of Voronoi tessellations to locate all individuals and alternatives in an attribute space. We provide an expression for the probability of correct match, and calculate, analytically and numerically, the average percentage of matches. We test dependence on the level of uncertainty and location. We find that the overall mismatch  is considerable even for low uncertainty—a possible concern for policy makers. We further explore a commonly used practice—allocating service representatives to assist individuals’ decisions. We show that within a given budget and uncertainty level, the effective allocation is for individuals who are close to the boundary between several Voronoi cells, but are not right on the boundary.

## Introduction

Important decisions people make, such as choosing health insurance, or choosing a school, require complex considerations. In many cases these considerations are further complicated by the uncertainty, or error of individuals in understanding what levels of specific attributes match their true preferences. For instance, in choosing a school, people might find it hard to specify what a “good” school means to them in terms of the level of specific attributes such as the number of Math hours, intensiveness of the music program, vocational training, geographic location, or whether the athletics program should include Quidditch^1^. After their children start attending the school, they might realize that they find the Math program less demanding, the music program too intensive, or that a 20 minute walk to school is more strenuous than they expected. Thus, they would have been happier with a school that has slightly different values on these attributes. Such patterns of post choice evaluation, regret, and disappointment have been empirically documented in the past literature (e.g. Westbrook^2^; Inman et al.^3^). Representing the relevant domain in the attribute space, we say that while individuals might *claim to know* where their preferences are located in the space, there is often uncertainty as to their *true desired location*. Only after the choice, they might realize that their perceived location doesn’t match their true needs and desires. This uncertainty might be a result of insufficient information about the meaning of different levels of attributes for them (e.g. what parental involvement, or an intensive music program require from them), or misconception as to what they really want.

In a market with several alternatives, such uncertainty might result in a mismatch—that is, choosing an alternative that is sub-optimal, although there are other available alternatives which better match one’s real needs. For example, while parents might be certain that they want the school with the intensive Math program, they might have actually been better off in a school with a less intensive program. Therefore their true preference would be in a slightly different location in the attribute space than what they initially thought they were. The choice literature indicates that mismatches happen when the choice task is complicated, or when individuals do not have enough previous experience with the specific choice task^4^.

Considerable effort is invested in reducing uncertainty to avoid mismatch in important decisions. Financial planners are used to consult in choosing health plans^5^, and advisors assist in pension plan choice^6^. Residents of major cities such as New York City employ expensive private consultants to assist in choosing a school^7^. From the perspective of the central planner that provides and supervises these services, too many mismatches are undesirable. A large group of dissatisfied service recipients might cause a decrease in the overall social welfare, which, in turn might lead to social and economic consequences. Assuming that the central planner wants to maximize the social welfare, as a goal by itself or in order to serve political and economic stability, it would better to minimize mismatches.

Our goal in this paper is to study the overall impact, from the perspective of the central planner, of decisions under the uncertainty described above (which we term hereafter as “uncertainty in preferences”). Similar to the school choice problem^8, 9^, the decision scenarios we model apply to high involvement, multiple attribute goods and services that are monitored by a central planner. They can be credence/experience goods and services, with a high importance for customer satisfaction and a high chance for post-choice evaluation and regret. While some of their attributes (such as distance or cost) might be very directional (a rational consumer will prefer zero distance and zero cost), many other attributes (e.g. level of religiousness, intensity of the Math program, hours of French per week etc.) are a matter of personal preference and can greatly vary between individuals. While the general formulation of the problem can incorporate a large number of market conditions and variables, we wish to work with a restrained set of conditions that will enable us to focus on the effect of uncertainty. Therefore, we focus on the case of no supply constraints, no specific market structure, and no interactions between individuals. Our modeling framework enables expansion to include these scenarios.

We use the representation of Voronoi tessellations to describe an attribute space with different alternatives, each having its attraction basin. Individuals can be also located in this space, according to their preference. Each individual has a perceived location, but since individuals might not correctly estimate the attribute levels that match their needs, this perceived location might be distant from their true preference, up to a certain uncertainty factor. The uncertainty creates an error in the perceived location of the individual, and hence can place the individual in the attraction basin of another, sub-optimal alternative, causing a mismatch.

We focus on the probability of correct match—that is, when the choice made is indeed the best alternative for this individual. We provide an expression for the probability for correct match, and show how it depends on the location in the attribute space and on the level of uncertainty. We give a formula for the average percentage of matches for low uncertainty level and use numerical simulation to extend the description for larger uncertainty.

We then extend our model by including a policy to help individuals obtain the correct decision and avoid mismatches. In some cases the central planner might offer “front-desk” services, which provide help through face-to-face or phone meetings. Such services are effective but costly. We use our model to study how the authority can allocate service representatives to individuals within a given budget in a way that will maximize the overall level of match.

Our contribution is by studying *decisions under uncertainty in preferences* from the perspective of the *central planner*. We draw inspiration from two streams of literature: Decisions under uncertainty, and Matching theory. Decisions under uncertainty have been mostly modeled from the individual’s point of view, and focused on the information search of individuals^10^, on how they sample the choice alternatives^11^, how they use social influence to compensate for the missing information^12^, and how they update their preferences based on each additional information bit they receive^13^. These models often consider factors such as expected utility from each alternative and risk aversion^14^. In choice modeling, random utility models were used to describe uncertainty in choice, under the assumption that some attributes are unobserved and are represented as random variables (e.g. Ben-Akiva and Lerman^15^), or, alternatively, that the decision-making individual considers each time only a subset of the attributes^16^. Works on post-choice evaluation (e.g. Inman et al.^3^) emphasized factors such as satisfaction and regret. This body of literature focuses on uncertainty in one’s understanding of the true value of the suggested alternatives, or, as in the random utility models, on cases where some dimensions of the attribute space are not taken into account during the choice. Our focus is on an attribute space and a set of alternatives that are entirely known to the individual, and the uncertainty in one’s understanding of his/her own needs and wants.

The implications of choice from the central planner’s perspective have mostly been studied without relating to uncertainty. Studies in matching theory^17, 18^ suggest algorithms for matching between individuals and outlets in various scenarios (schools, houses, hospital residency (see Sönmez and Ünver^19^ for review), where slots are limited, requiring one of the sides or both to rank their mutual preferences. Recent works on matching have begun to incorporate uncertainty in various forms: Ehlers and Massó^20^ describe a matching game where players are not sure about the preferences of other players. Hazon et al.^21^ study forecasting voting patterns, where the ranking of candidates for each voter is not fully known to an outside observer. Aziz et al.^22^ study the case where the individuals themselves are not certain in their rankings, but rather rank their preferences with a probability smaller than 1. These models are usually characterized by: (1) assuming limited capacity (otherwise all individuals get what they want); and (2) not having a direct access to the attributes, but rather to a rank ordering of alternatives. Their focus is to find the best matching algorithm that will create stable equilibrium.

Our modeling perspective draws from both streams—similar to the matching models we deal with matching alternatives to individuals, from the perspective of a central planner. Similar to the decision-under-uncertainty problems, our model deals directly with the attributes and does not use ranking of alternatives. However, we do not focus on the individual level, but rather look at the entire set of alternatives and individuals. We do not assume capacity constraints since, in the presence of uncertainty, mismatches can occur even without capacity limitations. Our focus is not empirical estimation, or efficient matching algorithm but rather to measure the probability for correct match and its dependence on various market factors. To the best of our knowledge, this work is the first to suggest a measure for the overall probability of matches, and calculate analytically its average value. Our model enables studying specific policies for minimizing the mismatch, such as the use of service representatives.

## The space of attributes and Voronoi tessellations

Our goal is to calculate the overall impact, from the perspective of the central planner, of decisions under uncertainty in preferences. To do so, we want to define a measure for the probability of a correct match for every possible individual preference, and then calculate its average value over a population. As explained above, most matching algorithms^19^ assume limited capacity, and the criterion for the optimal overall match is a stable equilibrium—that is, there is no pair of individuals who would be better-off by switching the alternatives they were assigned with. Therefore, these algorithms do not provide a continuous metric for the probability of a correct match. Individual level decision models that incorporated uncertainty^13, 15^ were used more for empirically estimating one’s utility and rarely provide an overall view of all the individuals and alternatives. The representation we seek is one that: (a) considers the entire attribute space and range of alternatives; (b) allows representation of the alternatives as well as the individuals; (c) provides a continuous measure of the match probability as a function of uncertainty; (d) can be easily expanded to incorporate interventions of the central planner, changes in the alternatives, and population changes.

To do so, we define a space ${{\mathscr{A}}}$ of attributes. Each dimension in this space is a numerical representation of a single attribute in the relevant context (e.g. level of religiousness, level of parental involvement, geographic location of the school). The space is a $K$ dimensional box with boundaries, representing the range of each attribute.

In this space we place *J* alternatives, (such as the various schools) giving to each alternative a point $P_j$ in this $K$-dimensional space.The location of an alternative represents its performance on each of the attributes. Each alternative has its attraction basin, and these partition the space of attributes ${{\mathscr{A}}}$ into a Voronoi tessellation^23, 24^.

The construction divides the space of attributes into Voronoi cells, which are the basins of attraction:

$$\begin{aligned} D_j=\{x\in {{\mathscr{A}}}: {\text{dist}}(x,P_j)\le {\text{dist}}(x,P_k), \quad \forall k =1,\dots , J \}. \end{aligned}$$

**Figure 1:**
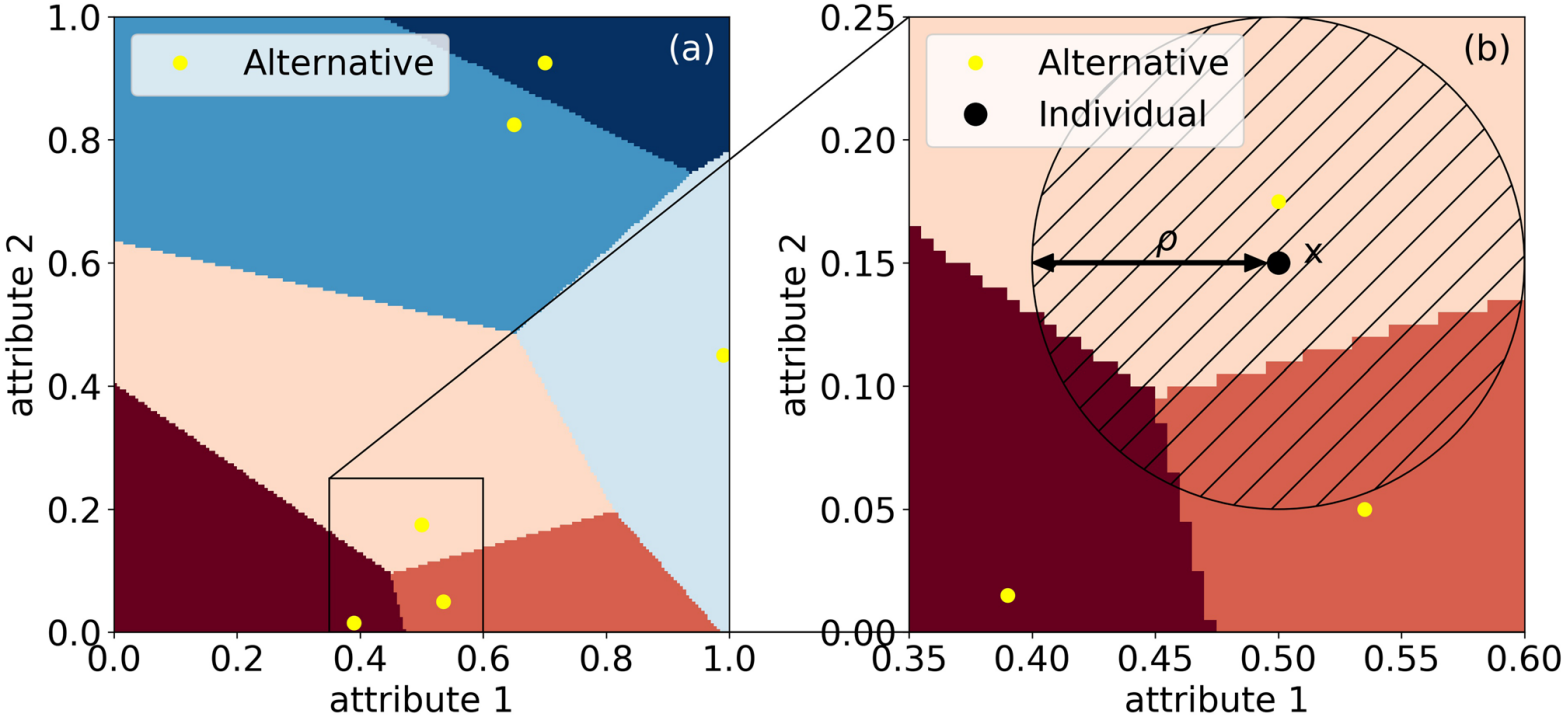
**Voronoi tessellation**. (**a**) An example for a two dimensional square $[0,1]^2$ of side length 1, where 6 alternatives (yellow) divide the area to distinct Voronoi cells. (**b**) In this example, $\rho = 0.1$. The probability $P_\rho (x)$ that an individual *x* (marked by the black dot) chose the correct Voronoi cell is the relative area of the part of the ball of radius $\rho$ around *x* which lies in the same Voronoi cell as *x*.

These are convex polyhedra, with disjoint interiors, whose union is all of ${{\mathscr{A}}}$, see Fig. 1a.

Individuals (say, the students, or their parents) are represented as points in the attribute space ${{\mathscr{A}}}$. The location of an individual *i* in this space, denoted by *x*, represents the true desire, or the “ideal” product of the individual (that is, a hypothetical alternative which should maximize individual *i*’s utility). It reflects both the desired level of attributes, as well as the importance of the each attribute to the individual. We want to match individuals to the alternative which most closely matches their preferences. A closest match would be an alternative $P_j$ so that the distance between the individual’s location $x\in {{\mathscr{A}}}$ is not greater than the distance to any other product, i.e., that resides within the same Voronoi cell. In utility terms, one can say that the utility derived from each actual alternative *j* can be represented as a function of the proximity of individual *i* to the location of alternative *j*.

We assume that the location of the alternatives in space is known to the individuals and is also known to the central planner. This is a reasonable assumption since consumers these days have wide access, through social media, customer reviews, and other online resources to the specifications of the alternatives in their choice set^25^.

### Modeling uncertainty

We add uncertainty to this representation: individuals, being sure they know what they want, locate themselves in a perceived place, which is distant from their true location in the attribute space up to an uncertainty factor $\rho$.

A common distinction is made in literature between uncertainty—which assumes the probability of each alternative is known, and ambiguity—where the individual also needs to assess the probability distribution from which the alternatives are drawn^26^. In this paper we do not deal with ambiguity. We assume that all alternatives are available and their properties are known. We describe uncertainty in the desired level of attributes, that is, uncertainty in preferences, and not in product location, product performance, or influence of uncontrolled factors.

Our setup has appeared in Computer Science, in the “nearest-neighbor search problem”, which returns the nearest neighbor of a query point *x* in a set of points ${{\mathscr{P}}}$ in ${{\mathbb{R}}}^d$. Both the data (the set of points ${{\mathscr{P}}}$) and the query (the point *x*) may be uncertain. For instance (see Beskales et al.^27^), in location-based services, a user may request the locations of the nearest gas stations. To protect the user’s privacy, an area that encloses the user’s actual location may be used as the query object, while gas stations (the data objects ${{\mathscr{P}}}$) have deterministic locations. In contrast to our goals, this literature focused on algorithmic and complexity aspects of the problem, see for instance the recent paper by Agrawal^28^ and the references there.

Due to the uncertainty $\rho$, an individual with a true location *x*, has a perceived location at a point around *x*. The perceived location is a point randomly drawn from a uniformly distributed ball of radius $\rho$ around the true location *x*. Note that neither the individual nor the central planner know the true location *x*. All they know is the perceived location. Even if individuals are aware of the uncertainty $\rho$, they can not reconstruct the drawing process. The uniformity assumption is required for the convenience of the formal analysis, and makes sense for a finite space and for the general case, where we assume zero information on the preferences.

Thus, rather than a point in the space of attributes, we actually have a ball $B(x,\rho )$ of all points at distance at most $\rho$ which define a possible perceived location of the individual whose true location is *x*. By taking the shape of a ball, we assume that the uncertainty is equal in all dimensions. This is a reasonable assumption for a general space, with no specific information on the dimension. However, even if the uncertainty is not equal in all dimensions, the uncertainty ball can be regarded as the circumscribed ball where $\rho$ is the uncertainty in the dimension with the maximal uncertainty.

Note, that while the uncertainty ball is uniform across attributes, and has a single radius for the entire population, the random draw of the perceived location generates heterogeneity across individuals: the perceived location is drawn for each and every individual separately, and therefore the *actual * error, namely, the distance between the perceived location and the true location varies across individuals and across attributes. The uncertainty $\rho$ can therefore be regarded as the maximum possible error in the perception.

The point *x* usually lies in a unique Voronoi cell $D_j$ which gives the correct match, while the ball $B(x,\rho )$ may intersect with some other cells.The probability $P_\rho (x)$ that the individual whose true location is the point *x* is assigned to the correct Voronoi cell to which it belongs (that is, the cases where the choice of the individual is indeed optimal) is the relative area (or volume) of the ball which lies in that cell, see Fig. 1b:

1 $$\begin{aligned} P_\rho (x) = \frac{{\text{vol}}(D_j\cap B(x,\rho ))}{{\text{vol}}B(x,\rho )} . \end{aligned}$$

As illustrated in Fig. 1, $P_\rho (x)$ strongly depends on the distribution of products in the attribute space, on the distance from the cell boundaries, and on the relationships between $\rho$ and the location within the Voronoi cell.

### The probability for correct match

From the perspective of the central planner which provides and supervises the services, a key measure of interest would be the effect of the uncertainty in individuals’ preferences on the overall mismatch for the entire population. A key measure we calculate is the *average* probability of correct match $\left\langle P_\rho \right\rangle$:

2 $$\begin{aligned} \left\langle P_\rho \right\rangle := \frac{1}{{\text{vol}}({{\mathscr{A}}})}\int _{{\mathscr{A}}}P_\rho (x)dx \end{aligned}$$

that is the average of $P_\rho (x)$ over the entire attribute space—all the locations *x*, and all the Voronoi cells $D_j$. We seek to describe its variation as we change the uncertainty factor $\rho$. For a uniformly distributed population in the attribute space $\left\langle P_\rho \right\rangle$ is given by:

3 $$\begin{aligned} \left\langle P_\rho \right\rangle = \frac{1}{{\text{vol}}({{\mathscr{A}}})}\sum _j \int _{D_j} \frac{\mathrm{vol}\left( B(x,\rho )\cap D_j\right) }{\mathrm{vol}B(x,\rho )}dx \end{aligned}$$

where $B(x, \rho )$ is the ball around *x* of radius $\rho$,and $\int _{D_j} dx$ means integration within a Voronoi cell *j* (see Supplementary Information Part 1 Proposition 1 for details).

**Figure 2:**
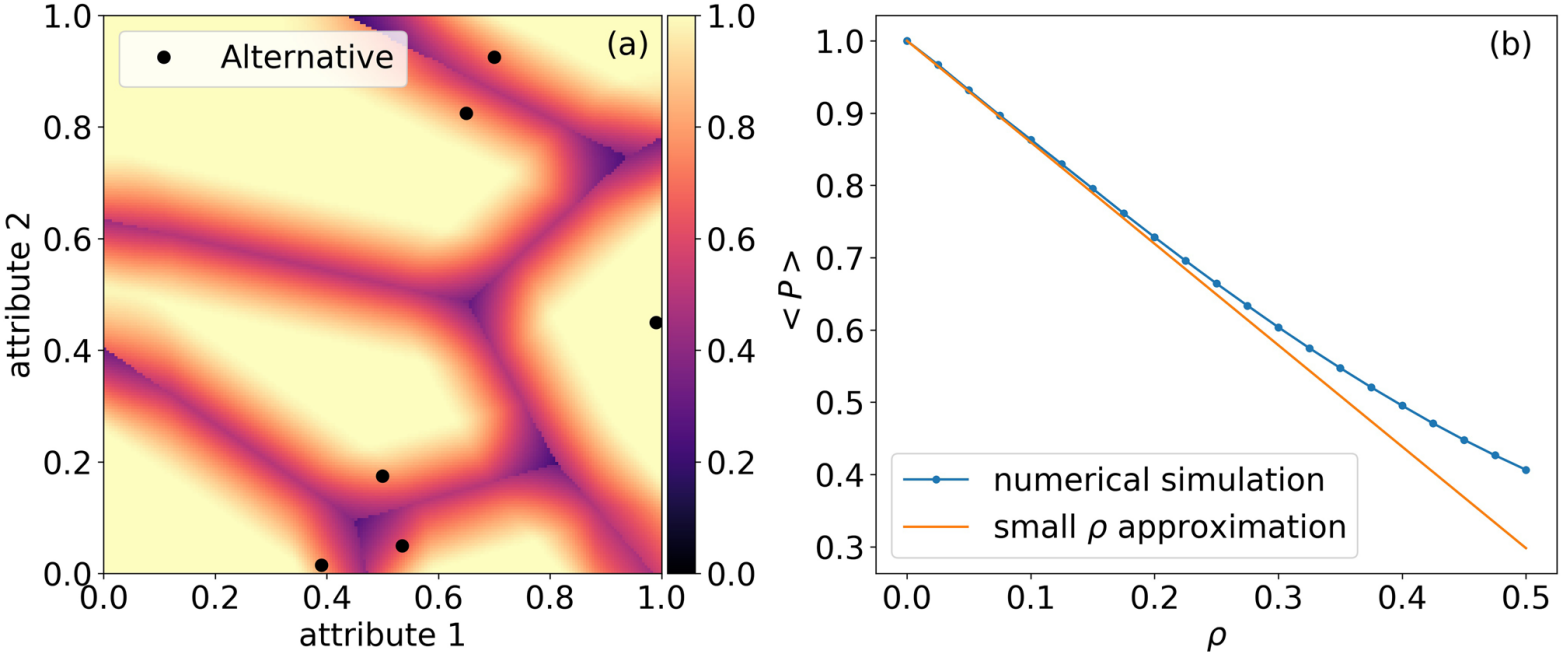
**Match probabilities**. (**a**) The local probability for a match, $P_\rho (x)$, is plotted as a color map for the example shown in Fig. 1. (**b**) Average probability for a match $\left\langle P_\rho \right\rangle$ as a function of $\rho$ for this configuration. Displayed is a comparison between the small $\rho$ linear approximation and the numerical calculation. The probability for correct match rapidly decreases with $\rho$ and the decrease is attenuated for large values of $\rho$, until saturation.

To provide an intuition as to how to compute this integral, recall that for a given individual in location *x*, when *x* is distanced more than $\rho$ to the boundary, a match will always be obtained. However, when *x* is closer to the boundary than $\rho$, the probability for a mismatch grows. As illustrated in Fig. 2a, for each cell, there is only a finite “danger zone”, around its boundaries, where a mismatch can occur. The cumulative area of the danger zones of all cells depends on two factors: (1) the size of $\rho$, (2) the total length of the boundaries between cells (in a general *K* dimensional space the danger zone will be the relevant volume, and the length will be in dimension $K-1$. In the one-dimensional case, where we only have one attribute, and the boundary consists of isolated points, the “length” of the boundary will be the number of points). For example, in Fig. 2a, describing a two dimensional space, this factor will be the total length of all the internal boundary segments between cells. When $\rho =0$, clearly $P_0\equiv 1$ as there is no uncertainty. When $\rho \gg 0$ is sufficiently large so that the uncertainty ball exceeds the combined size of the cells, the true location could be practically in any of the cells, meaning that the uncertainty is so vast that for every individual all the options seem reasonable to choose from.

Therefore, if $\rho$ is small enough to disregard overlap of danger zones from different cells, the volume of the total danger zone is approximately (to leading order) given by $\rho$ times the total area of the internal boundaries ($\partial ^{\mathrm{int}} D$). For the special case of a single attribute (one dimensional space), the attribute space is an interval of the size length(${{\mathscr{A}}}$), and the Voronoi cells are segments within this interval. The boundaries are single points, so the total area of the boundaries is given directly by the number of alternatives *J*. We give an analytic formula for $\left\langle P_\rho \right\rangle$ for the case of a small $\rho$ (namely, $\rho$ smaller or equal to half of the smallest segment (See Supplementary Information Part 1 Proposition 2):

4 $$\begin{aligned} \left\langle P_\rho \right\rangle = 1-\frac{J-1}{2} \frac{\rho }{{\text{length}}{{\mathscr{A}}}}. \end{aligned}$$

For higher dimensions $K\ge 2$, we compute the match probability for the *first variation* of $\left\langle P_\rho \right\rangle$, that is for the slope at $\rho =0$, which is the *v* in the expansion $\left\langle P_\rho \right\rangle \sim 1- v\rho$. The notation $f(\rho )\sim g(\rho )$ as $\rho \rightarrow 0$ means $\lim _{\rho \rightarrow 0} f(\rho )/g(\rho )=1$.

In dimension $K\ge 2$, the mean probability for correct assignment $\left\langle P_\rho \right\rangle$ for $\rho$ small is

5 $$\begin{aligned} \left\langle P_\rho \right\rangle \sim 1- \left( \frac{c_{K}}{{\text{vol}}{{\mathscr{A}}}} \sum _j {\text{vol}}_{K-1}(\partial ^{\mathrm{int}} D_j ) \right) \cdot \rho ,\quad \rho \searrow 0 \end{aligned}$$

where

6 $$\begin{aligned} c_{K} = \frac{1}{2} \frac{\Gamma \left( \frac{K}{2}+1\right) }{\sqrt{\pi } \Gamma \left( \frac{K+3}{2}\right) } = {\left\{ \begin{array}{ll} \frac{1}{\pi } \frac{2^{2m}}{(m+1)\left( {\begin{array}{c}2m+1\\ m\end{array}}\right) },&{} K=2m\;\mathrm{even} \\ \\ \frac{1}{2^{2m+2}}\left( {\begin{array}{c}2m+1\\ m\end{array}}\right) ,&K=2m+1\;\mathrm{odd}. \end{array}\right. } \end{aligned}$$

Here, $\Gamma$ is the Gamma function, thus $c_1=\frac{1}{4}$, $c_2=\frac{2}{3\pi }$, $c_3=\frac{3}{16}$, etc. See Supplementary Information Part 1 Proposition 3 for the proof. When $\rho$ is large, we can no longer disregard the overlap of the different danger zones, and we rely on numerical calculation of Eq. (3).

Note that in the special case of ($K=1$), Eq. (5) reduces to Eq. (4) as $\left\langle P_\rho \right\rangle =1- (\frac{1}{4}\sum _j\#\partial ^{\mathrm{int}} D_j ) \cdot \rho$, once we note that $c_K=\frac{1}{4}$. The boundary of an interior interval consists of 2 points, so that $\#\partial ^{\mathrm{int}} D_j=2$ for the $J-2$ interior intervals, and $\#\partial ^{\mathrm{int}} D_j=1$ for the two intervals at the boundary of the space—$j=1$, and $j=J$.

Equation (5) reveals the dominance of the cell boundaries on the match probability. It predicts that as $\rho$ increases, $\left\langle P_\rho \right\rangle$ decreases linearly, with a slope that depends strongly on the length of the boundaries between the different Voronoi cells.

To extend the above analysis for all the values of $\rho$ we numerically calculate $\left\langle P_\rho \right\rangle$ for the two dimensional case. We represent the market as a two dimensional grid, with 6 alternatives located as shown in Fig. 1 (the results are robust across location choices). We then execute three steps: first we assign for each grid point the best matched alternative. Second, we evaluate $P_\rho (x)$ by measuring the percentage of points having the same alternative in a sphere of radius $\rho$. Finally, we average $P_\rho (x)$ over the entire grid to obtain $\left\langle P_\rho \right\rangle$.

Figure 2a shows $P_\rho (x)$ for the setting described in Fig. 1, for $\rho$ = 0.075. While most of the attribute space enjoys a perfect probability for a match, near the boundaries the probability decreases. Panel b describes $\left\langle P_\rho \right\rangle$ as a function of $\rho$ for the same market configuration, comparing the small $\rho$ approximation to numerical calculations. The slope of $\left\langle P_\rho \right\rangle$ versus $\rho$ that is obtained from the approximation matches precisely the result of the numerical simulation. Both analytical and numerical calculations show that the probability for a correct match rapidly decreases with $\rho$. While for the approximation, the decrease is linear, the numerical simulations show that for large values of $\rho$, the decrease is attenuated, saturating at $\sum _{j=1}^J ({\text{vol}}D_j)^2$.

To illustrate the implications of the mismatch think of the opening example of choosing a school. In this setting, With $\rho =0.15$,  20% of the population will be dissatisfied, on average, with their choice, while there is another available school which matches their needs.

Note, that the matching in the above analysis is binary, that is, a mismatch happens when not assigning an individual with the true alternative, regardless of how the assigned alternative is close to the individual in the attribute space (this is an assumption in some of the literature on post-purchase evaluation e.g. Inman et al.^3^). In the Supplementary Information Part 2, we explore our results when the metric for the evaluation of the effect of uncertainty considers also the distance to the various alternatives.

#### Dependence on the number and distribution of alternatives

**Figure 3:**
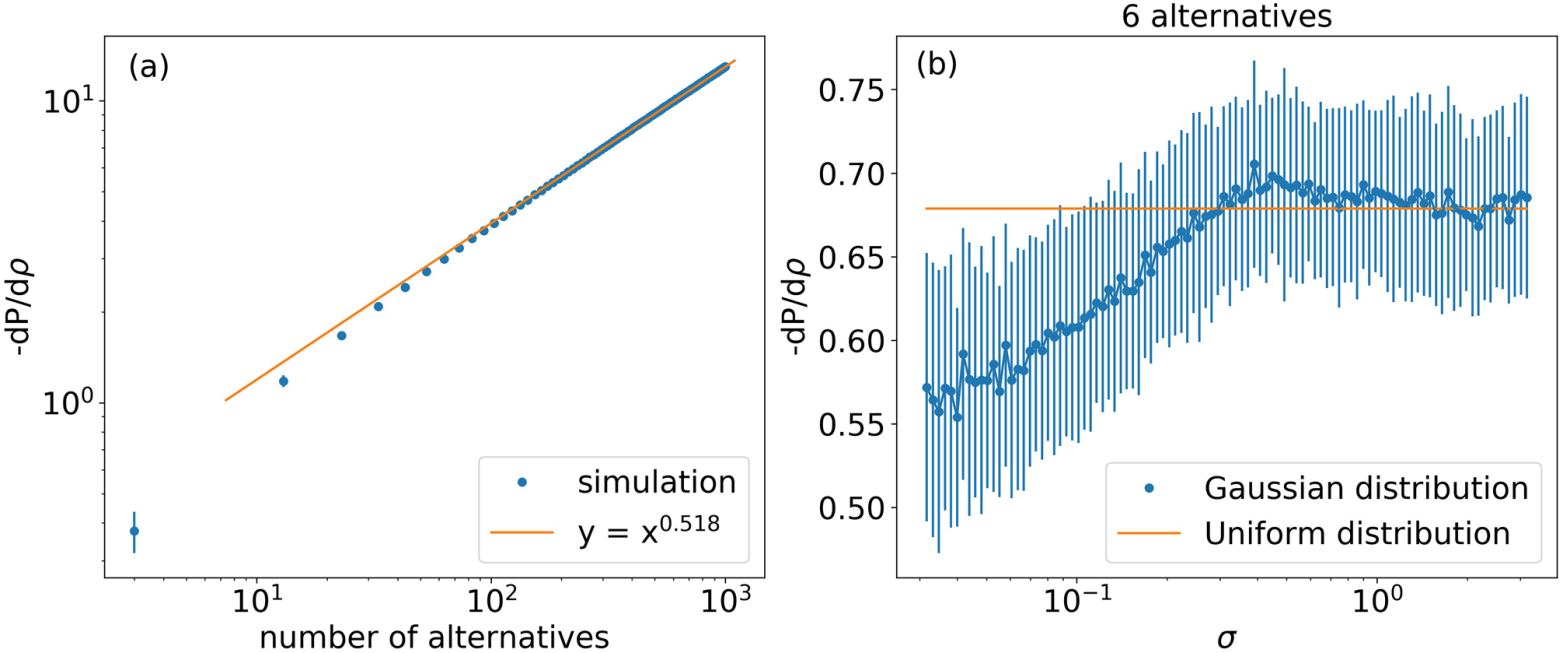
**Effects of the distribution and the number of alternatives on the probability for a match**. (**a**) $-d\left\langle P(\rho =0)\right\rangle / d\rho$ versus the number of alternatives. For each number of alternatives we generated 100 market configurations sampled from a uniform distribution. For each configuration we calculated the length of the boundaries between the resulting Voronoi cells and used Eq. (5) to compute $d\left\langle P(\rho =0)\right\rangle / d\rho$. We present the average value of the different configurations. The error bar shows the standard deviation. (**b**) Dependence on the distribution of alternatives: $-d\left\langle P(\rho =0)\right\rangle / d\rho$ versus $\sigma$, where $\sigma$ is the width of a trimmed Gaussian distribution, from which the location of alternatives is sampled. The simulation procedure is similar to panel (**a**). We vary $\sigma$ for the case of 6 alternatives (panel **b**).

The results shown in Fig. 2 provide an example for a specific configuration of six products. To assess the generalizability of this example we examined the effect of the number and distribution of the alternatives on the match probability. The slope of $\left\langle P_\rho \right\rangle$ where $\rho = 0$ serves as a useful metric, since it can be calculated directly from the length of boundaries. Higher slope indicates a stronger effect of the uncertainty on the match probability. Increasing the number of alternatives increases the slope—when more alternatives are available, the probability for a correct match decreases (see Fig. 3a). This might seem counter-intuitive, as one would expect that more alternatives to choose from imply greater overall possibilities for a match. However, at the same time, more options mean more probability for a mismatch—as an individual is surrounded by more alternatives, he is less likely to choose the optimal one . In our terminology, we say that the ball of uncertainty intercepts with a larger number of Voronoi cells. Note, that there is a body of literature on the relationship between the number of alternatives during choice process, and the level of satisfaction and regret. Having more choice alternatives to choose from often increases the difficulty of the task and reduces satisfaction (e.g. Schwartz^29^; Haynes^30^).

We further explore how the distribution of the alternatives in the attribute space affects the match probability. Assume the location of the alternatives is drawn from a trimmed Gaussian distribution with width $\sigma$. Figure 3b presents the slope $-d\left\langle P_\rho \right\rangle / d\rho \Big |_{\rho =0}$ versus $\sigma$ for a market with 6 alternatives. Increasing the width of the distribution increases the slope, thus reducing the probability for a correct match. The limiting case of uniform distribution has the lowest probability for a match (see Fig. 3b). The intuition behind this is that the more dense the alternatives are, they are more similar to each other, meaning that the effective number of real alternatives is small, which, as illustrated in panel a, implies a higher match probability.

### Allocating service representatives

The results described above indicate that under uncertainty in preferences, mismatches are very likely to occur and can affect a considerable portion of the population, which creates a challenge for the central planner. As explained above, the authorities often employ service representatives (reps, hereafter), which assist individuals in understanding their true needs through personal meetings. Thus, the central planner wishes to improve $\left\langle P_\rho \right\rangle$ by introducing meetings with reps, which once having met with an individual, improve the individual’s uncertainty from $\rho$ to a lower value $\rho _l<\rho$. Same as with the original uncertainty ball, our formulation practically allows heterogeneity in the amount of improvement: after the meeting with the service rep, a new perceived location is drawn, within a smaller radius $\rho _l$. The actual amount of improvement will naturally vary for each individual and each dimension. Thus, *ρ*_l_ is the upper bound for the possible improvement.  

Due to budget constraints these reps meet only a fraction *b* of the total population of individuals. We therefore ask who are the individuals which, within a given budget, should receive assistance from a rep in a way that will maximize the number of individuals who find their best matching alternative.

When the reps are allocated randomly, the new expected probability of correct assignment is

$$\begin{aligned} (1-b)\left\langle P_{\rho }\right\rangle +b\left\langle P_{\rho _l}\right\rangle \end{aligned}$$

Therefore, if we fix $\rho _l$ and $\rho$, and assuming that reps are randomly assigned to the population, increasing the proportion *b* of reps results in a *linear* increase of the expected probability of correct assignment.

We now check whether the central planner can improve the effectiveness of the reps by assigning them to specific individuals. To find the optimal assignment of service reps we define the local increase in match probability obtained from assigning a service rep to location *x* to be $\Delta (x,\rho ,\rho _l) = P_{\rho _l}(x)- P_{\rho }(x)$. Next, we choose *bN* grid points, where *N* is the total number of points on the grid, that have the maximal value of $\Delta (x,\rho ,\rho _l)$, and reduce the uncertainty at these points to be $\rho _l$. Finally, to calculate the improvement in the match probability obtained from this process, we average $P_\rho (x)$ over the entire grid. We note that this optimal allocation scheme uses the true location *x* of each individual, since we want to find the optimal allocation and spot the individuals who will have the maximum benefit from the service reps. In practice, as we stated above, *x* is not known to the central planner, and thus, the central planner’s implementation will be approximate, having its own error. We do not deal with such implementation error, but rather find the allocation which sets an upper limit to the benefit of the use of service reps.

**Figure 4:**
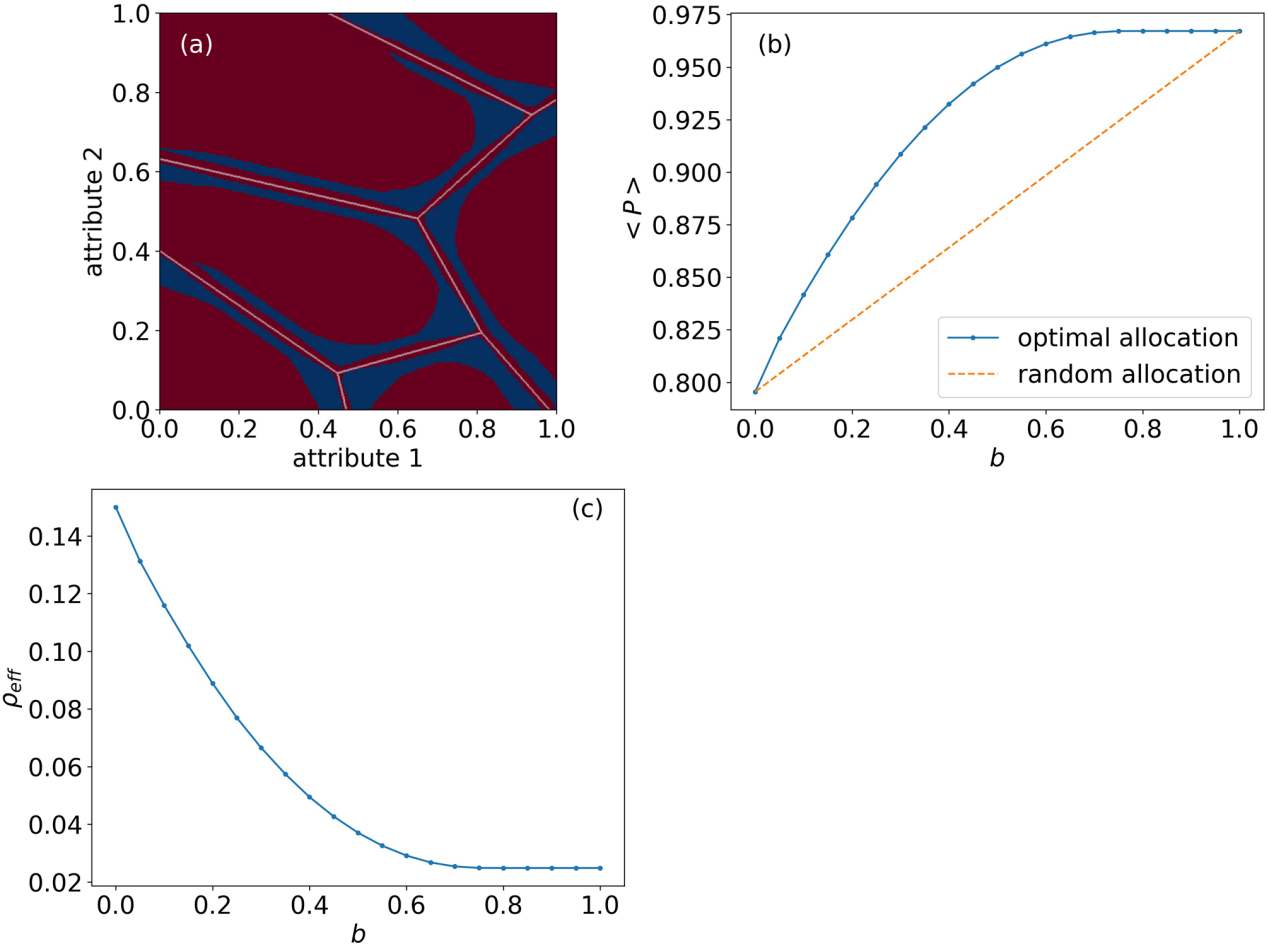
**Improving match probabilities using service reps**. (**a**) Areas which maximize the effectiveness of service reps (blue), within a budget $b=0.2$, for $\rho _l=0.025$ and $\rho =0.15$ (**b**) Average probability for a match $\left\langle P_\rho \right\rangle$ as a function of the budget *b*, where $\rho _l=0.025$ and $\rho =0.15$. The orange line shows a linear improvement when the reps are assigned randomly. The blue dots show maximal improvement when the reps are allocated optimally. (**c**) The equivalent overall $\rho$ which results in the same $\left\langle P_\rho \right\rangle$ as an optimal allocation of reps within a given budget *b*.

Figure 4 describes the overall improvement in $\left\langle P_\rho \right\rangle$ for various budget values *b*, where a budget is measured as the overall proportion of available rep meetings for the entire population. Panel a illustrates the areas which found to be optimal for receiving a meeting with the rep, within a budget $b=0.2$, for $\rho _l=0.05$ and $\rho =0.3$. We see that the places for optimal allocation (in blue), are those that are close to the boundaries between the Voronoi cells (white), but are not directly on the boundaries. When the distance from the boundary is smaller than $\rho _l$, meeting a rep will not significantly increase $\left\langle P_\rho \right\rangle$. Panel b presents $\left\langle P_\rho \right\rangle$ as a function of the budget *b*. While with random allocation, the improvement is linear with the budget, with the optimal allocation the curve shows a diminishing return and saturation at $b\approx 0.7$, meaning that the gain from allocating a service rep decreases as the number of allocated reps increases.

To further demonstrate the effectiveness of service reps, we compare two ways to increase the match probability: the first is allocation reps as discussed, and the second is reducing the overall uncertainty of the population through means such as educational or citizen involvement programs. Panel c shows, for each budget, what is the uncertainty $\rho$ that is equivalent to *b* percentage of the population meetings with reps. A budget that allows meeting reps for 20% of the population increases $\left\langle P_\rho \right\rangle$ from 0.8 to 0.88, which is equivalent to reducing $\rho$ for the entire population from 0.3 to 0.18. While in practice such a change in the entire population might require long term educational and citizen involvement programs, the same result could be obtained by providing a relatively simple, easy to operate, front-desk service to a pre-targeted population.

## Discussion

This paper deals with the overall impact of decisions, when individuals choose between alternatives, but have uncertainty as to the level of attributes that match their preferences.

We add to previous literature by suggesting a continuous measure for the probability of a correct match, in a modeling framework that considers the entire set of alternatives, attributes, and individuals, and can help central planners in designing their policies. We describe the attribute space as a Voronoi tessellation and use rigorous analysis and numerical simulations to describe the probability for correct match in space as a function of the uncertainty, and to calculate the average percentage of matches. We find that the overall mismatch can be considerable even for low levels of uncertainty, and thus can be a concern for policy makers. We further explore a practice often used by central planners—allocating service representatives to help individuals obtain the correct decisions. We use numerical simulations to show that within a given budget, the allocation is most effective for individuals whose preferences are at a certain distance from the boundaries of a Voronoi cell—not too deep in the cell, but yet not too close to a boundary.

This paper suggests several avenues for future research. First, one could re-examine our assumption on a uniform distribution of the population in the attribute space. Other distributions, such as bell-shaped distribution around a central value might diminish the impact of uncertainty (if, for example, there are several clusters of individuals and a single alternative is placed in the middle of each cluster), or alternatively enhance it (if preferences are centered around certain values, but the alternatives are scattered in space). An additional extension could be exploring the issue of capacity constraints—the scenario in which a mismatch could prevent *another* individual from being correctly matched. A third topic of interest would be endogenous sources of information, beside the reps, such as word-of-mouth from other users. Since this additional information also has uncertainty, it can hypothetically work in both directions and its influence on the reps allocation is not trivial.

## Supplementary information

Supplementary material 1
