# Supplementary material for: Modelling the expected probability of correct assignment under uncertainty
Source: Sci Rep. 2020 Sep 15;10:15080. doi: 10.1038/s41598-020-71558-x (PMC7493928; doi:10.1038/s41598-020-71558-x)
Supplement: Supplementary file 1 — Supplementary material 1 [file 41598_2020_71558_MOESM1_ESM.pdf]

MODELLING THE EXPECTED PROBABILITY OF CORRECT ASSIGNMENT UNDER UNCERTAINTY - SUPPLEMENTARY INFORMATION

TOM DVIR, RENANA PERES*, AND ZEÉV RUDNICK

** Corresponding author*

1. Proof of the formula for the first variation of the expected probability of correct assignment

We start with a space of attributes $\mathcal{A}$ which is a K-dimensional box, $\mathcal{A} = [a_1, b_1] \times [a_2, b_2] \times [a_K, b_K]$ with side-lengths $b_i - a_i$. We are given partition of the space of attributes $\mathcal{A}$ into Voronoi cells with disjoint interiors: $\mathcal{A} = \coprod_{j=1}^{J} D_j$. The cells are convex polytopes, so that the boundary of each cell is covered by finitely many hyperplanes.

We fix an uncertainty factor, a ball of radius $\rho > 0$, and for a point $x \in \mathcal{A}$ we ask what is the probability $P_\rho(x)$ that we assign the correct Voronoi cell, given this uncertainty factor ρ? That is, given that $x \in D_j$, what is the probability that we assign D_j as the basin of attraction, using an error bar of ρ? Note that the problem only makes sense for small ρ, because once ρ is sufficiently large so that the ball $B(x, \rho)$ covers all of $\mathcal{A}$, say $\rho > \rho_{\max}$, then the question is independent of ρ.

We can write a formula for the expected value $\langle P_\rho \rangle$ of $P_\rho(x)$ (as we average over x):

Proposition 1.

$$\langle P_\rho \rangle = \frac{1}{\operatorname{vol} \mathcal{A}} \sum_j \int_{D_j} \frac{\operatorname{vol}\Big(D_j \cap B(x,\rho)\Big)}{\operatorname{vol} B(x,\rho)} dx \tag{1}$$

where $B(x, \rho)$ is the ball around x of radius ρ.

If $\rho > \rho_{\max}$, then $\langle P_\rho \rangle$ saturates at $\langle P_\rho \rangle = \sum_{j=1}^{J} (\operatorname{vol} D_j)^2 / (\operatorname{vol} \mathcal{A})^2$.

Proof. To see (1), recall that given that $x \in D_j$, the probability $P_\rho(x)$ that we select D_j as the basin of attraction is the relative area of the intersection of the ball of radius ρ with the cell D_j:

$$P_\rho(x) = \begin{cases} \frac{\operatorname{vol}(D_j \cap B(x,\rho))}{\operatorname{vol} B(x,\rho)}, & x \in D_j \\ \\ 0, & x \notin D_j. \end{cases}$$

We want to compute the expected value of $P_\rho(x)$ (we average over x):

$$\langle P_\rho \rangle = \frac{1}{\operatorname{vol} \mathcal{A}} \sum_j \int_{D_j} P_\rho(x) dx = \sum_j \frac{1}{\operatorname{vol} \mathcal{A}} \int_{D_j} \frac{\operatorname{vol}\Big(D_j \cap B(x,\rho)\Big)}{\operatorname{vol} B(x,\rho)} dx.$$

To see the saturation value, take ρ to be larger than the diameter of the space of attributes $\mathcal{A}$, so that for each point x, the ball $B(x,\rho)$ coincides with all of $\mathcal{A}$. Then for each $x \in \mathcal{A}$, the intersection $B(x,\rho) \cap D_j = D_j$, and we can compute $\langle P_\rho \rangle$ simply as a conditional expectation, by writing

$$P_\rho(x) = \sum_{j=1}^{J} \mathbf{1}_{D_j}(x) \frac{\operatorname{vol} D_j}{\operatorname{vol} \mathcal{A}}$$

and then

$$\langle P_\rho \rangle = \frac{\int_{\mathcal{A}} P_\rho(x) dx}{\operatorname{vol} \mathcal{A}} = \frac{1}{\operatorname{vol} \mathcal{A}} \sum_{j=1}^{J} \int_{\mathcal{A}} \mathbf{1}_{D_j}(x) \frac{\operatorname{vol} D_j}{\operatorname{vol} \mathcal{A}} dx = \sum_{j=1}^{J} \Big(\frac{\operatorname{vol} D_j}{\operatorname{vol} \mathcal{A}} \Big)^2$$

as claimed. □

1.1. The one dimensional case.

Equation (1) makes sense in any dimension, but it is only in dimension $K = 1$, when the space of attributes is an interval $\mathcal{A} = [0, L]$, that we know how to extract an exact expression from it for small ρ.

Proposition 2. *For $K = 1$, and $\rho < \frac{1}{2} \min_j (a_{j+1} - a_j)$,*

$$\langle P_\rho \rangle = 1 - \frac{J-1}{2} \frac{\rho}{\operatorname{length} \mathcal{A}}.$$

Proof. We use equation (1): In this one-dimensional case, the Voronoi cells are intervals $D_j = [a_j, a_{j+1}]$, with $0 = a_1 < a_2 < \cdots < a_{J+1} = L$. We assume that

$$2\rho < \min_j \operatorname{length} D_j = \min_j (a_{j+1} - a_j).$$

To compute the contribution of each cell $D_j = [a_j, a_{j+1}]$, divide the region of integration into an interior region $D_j^{\text{int}} := [a_j + \rho, a_{j+1} - \rho]$ and two boundary regions $[a_j, a_j + \rho]$ and $[a_{j+1} - \rho, a_{j+1}]$.

For x in the interior region, we have $B(x,\rho) \subset D_j$ so that $D_j \cap B(x,\rho) = B(x,\rho)$ and hence

$$\begin{aligned}\int_{D_j^{\text{int}}} \frac{\operatorname{length}\Big(D_j \cap B(x,\rho)\Big)}{\operatorname{length} B(x,\rho)} dx &= \int_{D_j^{\text{int}}} 1 dx = \operatorname{length}(D_j^{\text{int}}) \\ &= (a_{j+1} - \rho) - (a_j + \rho) \\ &= a_{j+1} - a_j - 2\rho = \operatorname{length}(D_j) - 2\rho.\end{aligned}$$

To compute the contribution of the boundary components, note that there are two types, corresponding if they coincide with the boundary of the interval, namely $j = 1, J+1$, or not ($j = 2, \ldots, J$).

For components which do not intersect the boundary, namely if $j \neq 1, J+1$ then for all $x \in [a_j, a_j + \rho] \cup [a_{j+1} - \rho, a_{j+1}]$, the "ball" $B(x,\rho) = [x - \rho, x + \rho]$ has length 2ρ, but

$$B(x,\rho) \cap D_j = [x-\rho, x+\rho] \cap [a_j, a_{j+1}] = \begin{cases} [a_j, x+\rho], & a_j \le x \le a_j + \rho \\ [x-\rho, a_{j+1}], & a_{j+1} - \rho \le x \le a_{j+1} \end{cases}$$

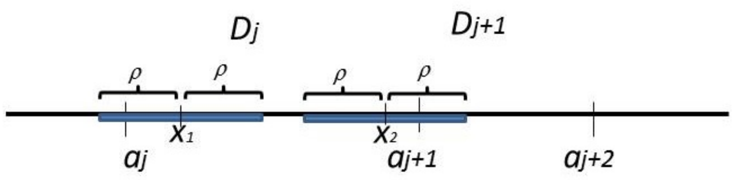

Supplementary Figure 1. The overlap of the "balls" $B(x,\rho) = [x-\rho, x+\rho]$ with the Voronoi intervals $D_j = [a_j, a_{j+1}]$.

(see Supplementary Figure 1) and

$$\operatorname{length} B(x,\rho)\cap D_j = \begin{cases} x+\rho-a_j, & a_j \le x \le a_j+\rho \\ a_{j+1}+\rho-x, & a_{j+1}-\rho \le x \le a_{j+1} \end{cases}$$

so that

$$\int_{a_j}^{a_j+\rho} \frac{\operatorname{length}\Big(D_j\cap B(x,\rho)\Big)}{\operatorname{length} B(x,\rho)}dx = \int_{a_j}^{a_j+\rho} \frac{x+\rho-a_j}{2\rho}dx = \frac{3}{4}\rho$$

and

$$\int_{a_{j+1}-\rho}^{a_{j+1}} \frac{\operatorname{length}\Big(D_j\cap B(x,\rho)\Big)}{\operatorname{length} B(x,\rho)}dx = \int_{a_{j+1}-\rho}^{a_{j+1}} \frac{a_{j+1}+\rho-x}{2\rho}dx = \frac{3}{4}\rho.$$

Altogether, we obtain for $j \neq 1, J$ that

$$\int_{D_j} p_\rho(D_j)(x)dx = \operatorname{length}(D_j) - 2\rho + \frac{3}{4}\rho + \frac{3}{4}\rho = \operatorname{length} D_j - \frac{1}{2}\rho.$$

For components which do intersect the boundary, that is for $D_1 = [0, a_2]$ or $D_J = [a_J, L]$, we have

$$B(x,\rho) = B(x,\rho)\cap D_1 = [0, x+\rho], \quad x \in D_1,$$

and

$$B(x,\rho) = B(x,\rho)\cap D_J = [x-\rho, L], \quad x \in D_J$$

so that

$$\frac{\operatorname{length} B(x,\rho)\cap D_j}{\operatorname{length} B(x,\rho)} = 1, \quad x \in D_1 \cup D_J$$

and we get a contribution of

$$\int_0^\rho 1dx = \rho = \int_{L-\rho}^L 1dx.$$

Therefore,

$$\int_{D_j} P_\rho(x)dx = \operatorname{length} D_j - 2\rho + \frac{3}{4}\rho + \rho = \operatorname{length} D_j - \frac{1}{4}\rho, \quad j = 1, J.$$

Altogether we find

$$\begin{aligned}\langle P_\rho\rangle &= \frac{1}{\operatorname{length}\mathcal{A}}\sum_{j=1}^{J}\int_{D_j} P_\rho(x)dx\\ &= \frac{1}{\operatorname{length}\mathcal{A}}\Big(\operatorname{length}D_1 - \frac{1}{4}\rho + \sum_{j=2}^{J-1}\Big(\operatorname{length}D_j - \frac{1}{2}\rho\Big) + \operatorname{length}D_J - \frac{1}{4}\rho\Big)\\ &= \frac{1}{\operatorname{length}\mathcal{A}}\Big(\sum_{j=1}^{J}\operatorname{length}D_j - \frac{J-1}{2}\rho\Big) = 1 - \frac{J-1}{2}\frac{\rho}{\operatorname{length}\mathcal{A}}\end{aligned}$$

as claimed. □

1.2. **Higher dimensions $K \geq 2$.** We now pass to the higher dimensional case $K \geq 2$. Our goal in this section is to obtain an exact formula for the *first variation* of $\langle P_\rho\rangle$, that is for the slope at $\rho = 0$.

For each Voronoi cell D_j, we denote by $\partial^{\text{int}}D_j$ the part of the boundary of D_j which does not lie on the boundary of the box (the space of attributes) $\mathcal{A}$.

Proposition 3. *In dimension $K \geq 2$, the mean probability for correct assignment $\langle P_\rho\rangle$ for ρ small is*

$$\langle P_\rho\rangle \sim 1 - \left(\frac{c_K}{\operatorname{vol}\mathcal{A}}\sum_j \operatorname{vol}_{K-1}(\partial^{\text{int}}D_j)\right)\cdot\rho, \quad \rho \searrow 0$$

where

$$c_K = \frac{1}{2}\frac{\Gamma\left(\frac{K}{2}+1\right)}{\sqrt{\pi}\,\Gamma\left(\frac{K+3}{2}\right)} = \begin{cases}\frac{1}{\pi}\frac{2^{2m}}{(m+1)\binom{2m+1}{m}}, & K = 2m \text{ even}\\[2ex] \frac{1}{2^{2m+2}}\binom{2m+1}{m}, & K = 2m+1 \text{ odd.}\end{cases} \tag{2}$$

Proof. We start by using equation (1)

$$\langle P_\rho\rangle = \frac{1}{\operatorname{vol}(\mathcal{A})}\sum_j\int_{D_j}\frac{\operatorname{vol}\left(B(x,\rho)\cap D_j\right)}{\operatorname{vol}B(x,\rho)}dx\ . \tag{3}$$

There are two types of points $x \in D_j$: Type I, those $x \in D_j$ so that the ball $B(x,\rho)$ is entirely contained in D_j, and type II are the rest (Supplementary Figure 2). Note that if x is close to the boundary of $\mathcal{A}$: $\operatorname{dist}(x,\partial\mathcal{A}) < \rho$, but far from the interior boundary of the cell, that is $\operatorname{dist}(x,\partial^{\text{int}}D_j) > \rho$, then $B(x,\rho) \subseteq D_j$ is entirely contained in the cell, even though it is only a truncated ball (Supplementary Figure 3). This means that these points are type I. Thus type II points are precisely those $x \in D_j$ so that $\operatorname{dist}(x,\partial^{\text{int}}D_j) < \rho$.

For type I points, we have $B(x,\rho)\cap D_j = B(x,\rho)$ so that the quotient of volumes equals unity:

$$\frac{\operatorname{vol}\left(B(x,\rho)\cap D_j\right)}{\operatorname{vol}B(x,\rho)} = 1, \quad x \text{ of type I.}$$

Thus the type I points contribute

$$\frac{1}{\operatorname{vol}(\mathcal{A})}\sum_j\int_{\substack{x\in D_j\\ x\text{ type I}}}\frac{\operatorname{vol}\left(B(x,\rho)\cap D_j\right)}{\operatorname{vol}B(x,\rho)}dx \qquad = \qquad \sum_j\frac{\operatorname{vol}\{x\in D_j \text{ type I}\}}{\operatorname{vol}(\mathcal{A})}. \tag{4}$$

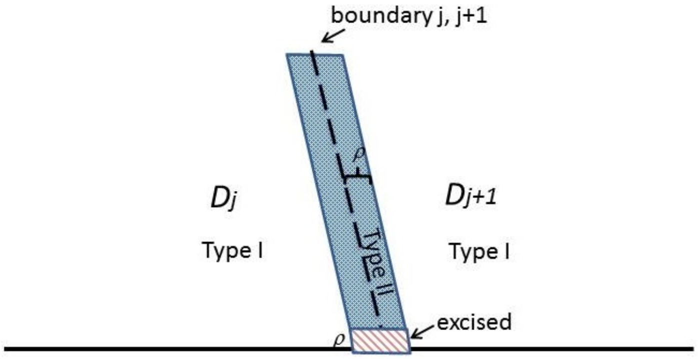

SUPPLEMENTARY FIGURE 2. Type I region, type II region (shaded) and the excised points near the boundary (shaded region with stripes).

The type II points are contained in a "strip" around the interior boundary of "width" 2ρ. We excise the contribution of points which are also ρ-close to $\partial\mathcal{A}$ or to more than one interior face (Supplementary Figure 2). The volume of these points is bounded by $O(\rho^2)$, since they are at distance $\leq \rho$ from the intersection of two faces or the intersection of a face with $\partial\mathcal{A}$, which has codimension 2. Since $\mathrm{vol}\left(B(x,\rho)\cap D_j\right)/\mathrm{vol}B(x,\rho) \leq 1$ in any case, the total contribution of such points is $O(\rho^2)$, which is negligible. Thus we need only consider points x with $\mathrm{dist}(x, \partial^{\mathrm{int}} D_j) < \rho$ and in addition that $B(x,\rho)$ is an actual Euclidean ball, not a truncated one.

Proposition 4. *For ρ sufficiently small, the contribution of type II points is*

$$\sum_j \frac{\mathrm{vol}(x \in D_j \text{ type II})}{\mathrm{vol}(\mathcal{A})} - \frac{c_K\, \mathrm{vol}_{K-1}\, \partial^{\mathrm{int}} D_j}{\mathrm{vol}(\mathcal{A})}\rho + O(\rho^2).$$

Putting together equation (4) and Proposition 4 gives Proposition 3. □

1.3. **Proof of Proposition 4.** Fix a component H of the interior boundary $\partial^{\mathrm{int}} D_j$; H is a hyperplane. After rotation, reflection and translation of the diagram, we may assume that the boundary component H is the coordinate hyperplane $H = \{x = (x_1, \ldots x_K) : x_K = 0\}$, and that the cell D_j lies in the top half-space $H_+ = \{(y_1, \ldots, y_K) : y_K \geq 0\}$ (Supplementary Figure 3). Then for every $x \in H_+$, we have $\mathrm{dist}(x, H) = x_K$ and we assume that $0 \leq x_K \leq \rho$. We need to compute

$$\int\limits_{x:(x_1,\ldots,x_{K-1},0)\in H} \frac{\mathrm{vol}\left(B(x,\rho)\cap D_j\right)}{\mathrm{vol}B(x,\rho)} dx_1 \ldots dx_K$$

$$= \frac{1}{\mathrm{vol}B(0,\rho)} \int\limits_{\substack{0\leq x_K\leq\rho \\ (x_1,\ldots,x_{K-1},0)\in H}} \mathrm{vol}\left(B(x,\rho)\cap D_j\right) dx_1 \ldots dx_K.$$

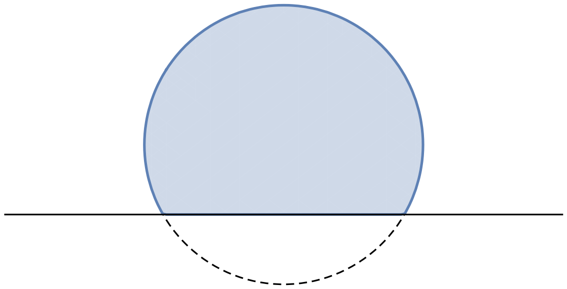

Supplementary Figure 3. A truncated ball

We fix the first $K-1$ components $x^0 = (x_1, \ldots, x_{K-1})$, and compute the integral over x_K:

Lemma 5. *Fix* $(x_1, \ldots, x_{K-1})$ *so that* $(x_1, \ldots, x_{K-1}, 0) \in H$. *Then*

$$\frac{1}{\operatorname{vol} B(0,\rho)} \int_{x_K=0}^{\rho} \operatorname{vol}\Big(B\Big((x_1, \ldots, x_{K-1}, x_K)\, , \rho\Big) \cap H_+\Big) dx_K$$
$$= 1 - c_K \cdot \rho + O(\rho^2).$$

Proof. Since the integral is independent of the first $K-1$ components, those may be taken to be zero, so that $(x_1, \ldots, x_{K-1}) = (0, \ldots, 0)$. So we want to compute

$$\int_{x_K=0}^{\rho} \operatorname{vol}\Big(B\Big((0, \ldots, 0, x_K)\, , \rho\Big) \cap H_+\Big) dx_K.$$

The set $B\Big((0, \ldots, 0, x_K)\, , \rho\Big) \cap H_+$ is the bigger half of the ball $B\Big((0, \ldots, 0, x_K)\, , \rho\Big)$ (see Supplementary Figure 3); we find it easier to compute the integral over the complementary, smaller half, which is a spherical cap (Supplementary Figure 4), and this in turns equals

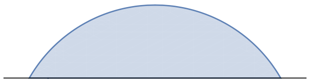

Supplementary Figure 4. A spherical cap

$$\int_{x_K=0}^{\rho} \operatorname{vol}\Big(B\Big((0, \ldots, 0, x_K)\, , \rho\Big) \cap H_+\Big) dx_K$$
$$= \operatorname{vol} B(0,\rho) - \int_{x_K=0}^{\rho} \operatorname{vol}\Big(B\Big((0, \ldots, 0, -x_K)\, , \rho\Big) \cap H_+\Big) dx_K.$$

Dividing by $\operatorname{vol} B(0,\rho)$ gives

$$1 - \frac{1}{\operatorname{vol} B(0,\rho)} \int_0^{\rho} A(x_K) dx_K$$

where $A(x_K)$ is the volume of the small spherical cap

$$\begin{aligned}
A(x_K) &:= \mathrm{vol}\Big(B\Big(\,(0,\dots,0,-x_K)\,,\rho\Big)\cap H_+\Big)\\
&= \mathrm{vol}\{(\vec{y},z) : \vec{y}\in\mathbb{R}^{K-1},\ z\geq 0,\ |\vec{y}|^2+(z+x_K)^2\leq\rho^2\}\\
&= \int_{\substack{\vec{y}\in\mathbb{R}^{K-1}\\ |\vec{y}|^2\leq\rho^2-x_K^2}}\int_{z=0}^{-x_K+\sqrt{\rho^2-|\vec{y}|^2}} dz d\vec{y}\\
&= \int_{|\vec{y}|^2\leq\rho^2-x_K^2}\Big(\sqrt{\rho^2-|\vec{y}|^2}-x_K\Big)d^{K-1}\vec{y}\\
&= \int_{|\vec{y}|^2\leq\rho^2}\mathbf{1}(|\vec{y}|^2+x_K^2\leq\rho^2)\Big(\sqrt{\rho^2-|\vec{y}|^2}-x_K\Big)d^{K-1}\vec{y}.
\end{aligned}$$

Now integrate over $x_K\in[0,\rho]$: Switching order of integration gives

$$\begin{aligned}
\int_0^\rho A(x_K)dx_K &= \int_0^\rho\int_{|\vec{y}|^2\leq\rho^2}\mathbf{1}(|\vec{y}|^2+x_K^2\leq\rho^2)\Big(\sqrt{\rho^2-|\vec{y}|^2}-x_K\Big)d^{K-1}\vec{y}dx_K\\
&= \int_{|\vec{y}|^2\leq\rho^2}\int_{x_K=0}^{\rho}\Big(\sqrt{\rho^2-|\vec{y}|^2}-x_K\Big)\mathbf{1}(|\vec{y}|^2+x_K^2\leq\rho^2)dx_Kd^{K-1}\vec{y}\\
&= \int_{|\vec{y}|^2\leq\rho^2}\int_{x_K=0}^{\sqrt{\rho^2-|\vec{y}|^2}}\Big(\sqrt{\rho^2-|\vec{y}|^2}-x_K\Big)dx_Kd^{K-1}\vec{y}\\
&= \int_{|\vec{y}|^2\leq\rho^2}(\rho^2-|\vec{y}|^2)d^{K-1}\vec{y}-\int_{|\vec{y}|^2\leq\rho^2}\frac{1}{2}(\rho^2-|\vec{y}|^2)d^{K-1}\vec{y}\\
&= \frac{1}{2}\int_{|\vec{y}|^2\leq\rho^2}(\rho^2-|\vec{y}|^2)d^{K-1}\vec{y}\\
&= \rho^{K+1}\frac{1}{2}\int_{|\vec{y}|\leq 1}(1-|\vec{y}|^2)d^{K-1}\vec{y}.
\end{aligned}$$

When $K=2$, this equals

$$\rho^3\frac{1}{2}\int_{y=-1}^{1}(1-y^2)dy=\frac{2}{3}\rho^3$$

and dividing by the area of $B(0,\rho)=\pi\rho^2$ gives

$$\frac{1}{\mathrm{area}\,B(0,\rho)}\int_0^\rho A(x_2)dx_2=\frac{2}{3\pi}\rho=c_2\rho.$$

For $K\geq 3$, we will use polar coordinates: In $G\geq 2$ dimensions (we will take both $G=K-1$ and $G=K$)

$$x_j=r\cos(\theta_j)\prod_{k=1}^{j-1}\sin\theta_k,\quad j=1,\dots,G-1,\quad x_G=r\prod_{k=1}^{G-1}\sin\theta_k$$

with $r\geq 0$, $0\leq\theta_j\leq\pi$ for $j=1,\dots,G-2$ and $0\leq\theta_{G-1}\leq 2\pi$. The Jacobian of this transformation is

$$J_G(r,\theta)=r^{G-1}\prod_{j=1}^{G-2}(\sin\theta_j)^{G-1-j}.$$

The volume of the ball $B(0,\rho) \subset \mathbb{R}^K$ in dimension $G = K$ is thus

$$\begin{aligned}
\mathrm{vol}_K\, B(0,\rho) &= \int_{r=0}^{\rho} r^{K-1}dr \prod_{j=1}^{K-2} \int_{\theta_j=0}^{\pi} (\sin\theta_j)^{K-1-j} d\theta_j \int_{\theta_{K-1}=0}^{2\pi} d\theta_{K-1} \\
&= \rho^K \frac{2\pi}{K} \int_{\theta_1=0}^{\pi} (\sin\theta_1)^{K-2} d\theta_1 \cdot \prod_{j=2}^{K-2} \int_0^{\pi} (\sin\theta_j)^{K-1-j} d\theta_j \\
&= \frac{2\pi}{K} \int_{\theta_1=0}^{\pi} (\sin\theta_1)^{K-2} d\theta_1 \cdot \prod_{i=1}^{K-3} \int_0^{\pi} (\sin\theta_j)^{K-2-i} d\theta_i \cdot \rho^K \\
&= \frac{2\pi}{K} \frac{\sqrt{\pi}\Gamma\left(\frac{K-1}{2}\right)}{\Gamma\left(\frac{K}{2}\right)} \cdot \prod_{i=1}^{K-3} \int_0^{\pi} (\sin\theta_j)^{K-2-i} d\theta_i \cdot \rho^K.
\end{aligned}$$

Using polar coordinates in $\mathbb{R}^{K-1}$, $K \geq 3$ (so that $G = K-1$), gives

$$\begin{aligned}
\int_{|\vec{y}|\leq 1} (1-|\vec{y}|^2) d^{K-1}\vec{y} &= \int_0^1 (1-r^2) r^{K-2} dr \prod_{j=1}^{K-3} \int_0^{\pi} (\sin\theta_j)^{K-2-j} d\theta_j \int_0^{2\pi} d\theta_{K-2} \\
&= \frac{2}{K^2-1} 2\pi \prod_{j=1}^{K-3} \int_0^{\pi} (\sin\theta_j)^{K-2-j} d\theta_j
\end{aligned}$$

so that

$$\int_0^{\rho} A(x_K) dx_K = \frac{1}{K^2-1} 2\pi \prod_{j=1}^{K-3} \int_0^{\pi} (\sin\theta_j)^{K-2-j} d\theta_j \cdot \rho^{K+1}.$$

Dividing we find that

$$\frac{1}{\mathrm{vol} B(0,\rho)} \int_0^{\rho} A(x_K) dx_K = \frac{K}{K^2-1} \frac{\Gamma\left(\frac{K}{2}\right)}{\sqrt{\pi}\Gamma\left(\frac{K-1}{2}\right)} \cdot \rho = \frac{\Gamma(\frac{K}{2}+1)}{2\sqrt{\pi}\Gamma(\frac{K+3}{2})} \cdot \rho$$

which equals $c_K \rho$. □

We can now complete the proof of Proposition 4: Until now, we have fixed the coordinates $(x_1, \ldots, x_{K-1})$, where the particular face of the cell is $(x_1, \ldots, x_{K-1}, 0) \in H \cap D_j$; integrating over these coordinates, we obtain the $(K-1)$-dimensional volume of that face up to an error of $O(\rho^2)$, and summing over all interior faces of the cell D_j and then over the various cells, we obtain

$$\frac{1}{\mathrm{vol}\,\mathcal{A}} \sum_j \Big(\mathrm{vol}(x \in D_j :\ \text{of type II}) - \mathrm{vol}_{K-1}(\partial^{\mathrm{int}} D_j) c_K \rho \Big) + O(\rho^2)$$

as asserted by Proposition 4. □

2. Distance Based Matching Metric

We explore the sensitivity of our results in Figure 2 of the main text to a matching measure which is based on distance rather than a matching/non-matching binary classification. One can say, that a binary match/non-match classification does not provide information as to how much the chosen alternative is worse than the optimal one, and thus it makes it harder to evaluate the overall dissatisfaction in the population. A metric which measures the average distance between the possible

chosen alternatives and the true location, might provide additional information as to the level of satisfaction of the individual from the chosen alternative.

To construct such metric, consider an individual at location x, within a single Voronoi cell D_j. If there is no uncertainty in the perceived location of that individual ($\rho = 0$),the individual is assigned to alternative j, at a distance $d(\rho = 0, x) = |x - j|$ to the alternative. If the individual mistakenly perceives his position as y, leading to choosing another alternative, i, then the distance between the true location and the chosen alternative is $|x - i|$. Assuming a uniformly distributed error ball of radius ρ around x, we obtain the average distance between the chosen alternative and the true position as:

$$d\left(\rho, x\right) = \sum_j \frac{\mathrm{vol}\left(D_j \cap B(x, \rho)\right)}{\mathrm{vol}\, B(x, \rho)} \left|x - j\right| \tag{5}$$

Supplementary Figure 5a shows the effect of uncertainty on the average distance to the chosen alternative: $d\left(\rho, x\right) - d\left(\rho = 0, x\right)$. By integrating over the attribute space we obtain the average distance between all of the individuals and their chosen alternatives. To measure the elasticity of the overall match on the error ρ, we divide the above average by the average distance obtained for $\rho = 0$:

$$\left\langle d\left(\rho\right)\right\rangle = \frac{\int d\left(\rho, x\right) dx}{\int d\left(\rho = 0, x\right) dx} \tag{6}$$

The metric $\left\langle d\left(\rho\right)\right\rangle$ represents the average incremental distance between the true location and all the possible chosen alternatives within the error ball, relative to the no error case. The larger is its deviation from 1, the higher is the distance between the individuals and their chosen alternatives.

Supplementary Figure 5a visualizes the effect of uncertainty for the case of the attribute space shown in Figures 1 and 2 of the main text. We plot $d(\rho, x) - d(\rho = 0, x)$ at each point of the attribute space. Just as in the case of the binary metric, most of the effect of the uncertainty lies within a strip of radius ρ around the boundaries. However, unlike the binary metric, where the boundaries are the most sensitive to the occurrence of a mismatch, for the distance-based metric, the boundaries are the regions which are the least sensitive to a mismatch, as the distance to the alternatives on both sides of the boundary is of similar magnitude.

Supplementary Figure 5b shows $1/\left\langle d\left(\rho\right)\right\rangle$ vs. ρ for the same attribute space. The value of $1/\left\langle d\left(\rho\right)\right\rangle$ decreases with ρ. Note, that unlike the linear decrease in the binary metric, this decrease, for low values of ρ, can be fitted by a parabola $\left\langle d\left(\rho\right)\right\rangle \propto \rho^2$. The effect of the uncertainty is thus second order in ρ.

To compare the effect of the uncertainty between the binary and the distance based cases, consider $\rho = 0.15$. The mismatch probability in the binary case is 20% (as shown in Figure 2 of the main text), however the value of $\langle d(\rho)\rangle$ is 1.03. That is, although on average 20% of the population is expected to choose an alternative which is not optimal, the distance to their chosen alternatives is expected to increase by 3%.

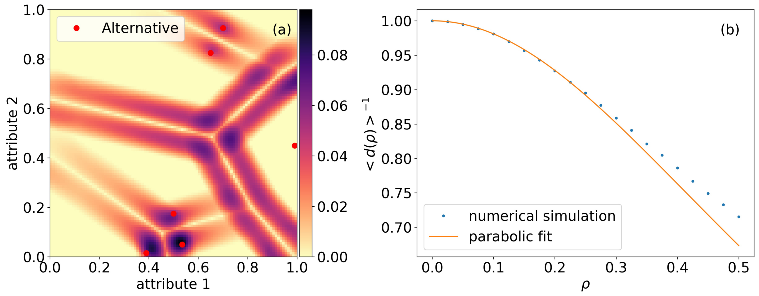

Supplementary Figure 5. **Distance based matching. a.** The effect of the uncertainty on the average distance to the chosen alternative: $d(\rho, x) - d(\rho = 0, x)$ obtained using numerical simulations for the example shown in Figure 1 of the main text and $\rho = 0.125$ **b.** $1/\langle d(\rho)\rangle$ vs. ρ for the same example.
